# Interference patterns in ionization of Kramers–Henneberger atom

**DOI:** 10.1038/s41598-022-21549-x

**Published:** 2022-10-11

**Authors:** I. A. Ivanov, A. S. Kheifets, Kyung Taec Kim

**Affiliations:** 1 Center for Relativistic Laser Science, Institute for Basic Science, Gwangju, 61005 Republic of Korea; 2 Research School of Physics, Australian National University, Canberra, ACT 2601 Australia; 3 Department of Physics and Photon Science, Gwangju Institute of Science and Technology, Gwangju, 61005 Republic of Korea

**Keywords:** Atomic and molecular interactions with photons, Attosecond science

## Abstract

We combine IR pump and XUV probe laser pulses to visualize the Kramers–Henneberger (KH) state of the potassium atom. We demonstrate that ionization of such an atom exhibits some molecular-like features such as low order interference maxima in photoelectron momentum spectra. The locations of these maxima allow to estimate spatial dimensions of the KH atom and can be used for accurate calibration of high intensity laser fields. At the same time, we show that an analogy between the KH atom and a homo-nuclear diatomic molecule cannot be extended too far. In particular, higher order interference maxima are very difficult to observe in the case of the KH state. We attribute this to a particular structure of the KH potential which does not confine electron motion to a well-defined potential well unlike in real diatomic molecules.

## Introduction

The formation of the Kramers–Henneberger (KH) state in an atom exposed to a strong laser field has been a subject of numerous investigations. In a seminal work^1^ Henneberger showed that the ionic potential, viewed from the electron reference frame and averaged over its rapid oscillations, can actually bind the electron into a stable KH atom. This binding is thought to be behind the process of laser driven stabilization^2^. Various techniques have been proposed to image the KH atom. One such imaging is offered by means of photoelectron spectroscopy^3^. The bound states of the KH atom leave their distinct marks in the photoelectron momentum distribution (PMD). Even more remarkably, a bound state of the KH atom can be distorted into a dichotomic molecular-like state^2,4^ whose PMD displays a double-slit interference pattern^5^. This pattern is suggested to serve as an unambiguous evidence of the KH states leading to adiabatic stabilization. In addition, the authors of the Ref.^5^ claim that dichotomic hydrogen atom displays a number of other molecular peculiarities, such as charge-resonance enhanced ionization and electron spin flipping.

Inspired by these findings, we carry out a systematic investigation of the PMD of the KH atom. The point of departure from the earlier work^5^ is our use of an infrared (IR) laser pump and an extreme ultraviolet (XUV) probe. These pump and probe pulses were reverted in the previous work. As the KH atom is driven more strongly by a low frequency field, the use of an IR pump is more efficient in comparison with an XUV one. While the hydrogen atom was used in the Ref.^5^, we use the potassium atom with a smaller ionization potential. Thus, the formation of the KH state is easier to observe. It can be achieved in weaker IR fields and probed by lower intensity XUV pulses. The probe process requires absorption of a single XUV photon and can be conveniently described within the lowest order perturbation theory (LOPT).

As in the previous work^5^, our simulations reveal molecular-like structures in PMD of the KH atom such as low order interference maxima. The location of these maxima can be used to estimate a spatial dimension of the KH atom. However, an analogy between the KH atom and a homo-nuclear diatomic molecule is not complete. In particular, higher order interference maxima are missing in the PMD. These maxima should be amply present in a molecule with a comparable spatial extent. We attribute their absence in the KH atom to a particular structure of the KH potential which does not confine electron motion to a well-defined potential well.

Our paper is structured in the following way. In the “Theory” section we introduce the KH potential and present our numerical technique used to describe the KH state of the potassium atom. We further introduce the XUV probe and describe single-photon ionization of the KH state. In the “Results and discussion” section we show and analyze our numerical results for the PMD’s. The concept of the information theoretic Shannon entropy is used to characterize the sharpness of the KH state in the coordinate and momentum spaces. We demonstrate that there is a profound difference between the electron localization in the KH state and a diatomic molecule. We show that, despite this fact, we can still extract some information about spatial extent of the KH state from the interference patterns in the PMD’s. This information can be employed for an accurate calibration of the high intensity electromagnetic fields. We conclude in the “Conclusion” section by summarizing our major findings.

Atomic units (a.u.) are used throughout the paper.

## Theory

### KH atom description

The formation of the KH atom is described in the literature^1,3,6^. We follow these works and consider a one-electron atom with an effective atomic potential $V({\varvec{r}})$, and subject it to a monochromatic linearly polarized laser field ${\varvec{E}}(t)={\varvec{e}}_x E_0 \cos {\omega t}$. We merge the origin of our reference frame with the atomic electron oscillating in the field. This is achieved by performing a canonical transformation generated by the operator

1 $$\begin{aligned} \hat{F}= \exp {\left\{ i\int {\varvec{A}}(\tau )\cdot \hat{\varvec{p}}\ d\tau \right\} }, \end{aligned}$$

where $\displaystyle {\varvec{A}}(t)=-\int \limits _0^t {\varvec{E}}(\tau )\ d\tau$ is the field vector potential. This way we transform the initial velocity gauge Hamiltonian to the so-called Kramers–Henneberger gauge, where the Hamiltonian of the system can be written as:

2 $$\begin{aligned} \hat{H}= {\hat{\varvec{p}}^2\over 2}+ V_\mathrm{atom}({\varvec{r}}+ a_0\hat{\varvec{e}}_x\cos {\omega t}), \end{aligned}$$

with $a_0= E_0 \omega ^{-2}$ being the excursion radius of an electron in the monochromatic field $E_0 \cos {\omega t}$. By expanding further this expression in a Fourier series and keeping only the constant term^3^, we obtain the Hamiltonian $\hat{H}_\mathrm{KH}$ describing the KH atom:

3 $$\begin{aligned} \hat{H}_\mathrm{KH} = {\hat{\varvec{p}}^2\over 2}+ V_\mathrm{KH}({\varvec{r}}). \end{aligned}$$

Here the KH potential $V_\mathrm{KH}({\varvec{r}})$ is defined as a cycle average

4 $$\begin{aligned} V_\mathrm{KH}({\varvec{r}})= {1\over 2\pi } \int \limits _0^{2\pi } V[{\varvec{r}}+ {\varvec{e}}_x \alpha (\phi )]\ d\phi , \end{aligned}$$

with $\displaystyle \alpha (\phi )= a_0\cos {\phi }$. The Hamiltonian (3) of the KH atom is, therefore, obtained by neglecting all the harmonics except the zero order one in the Fourier expansion of the Hamiltonian (2). Whether keeping the constant term and neglecting all the oscillatory terms provides a good description of the quantum dynamics depends on the particular laser and atomic system parameters. For higher driving laser frequencies in the UV and XUV ranges, such an approximation is reminiscent of the well-known fast oscillations averaging method in classical mechanics^7,8^. The quantum-mechanical version of such a method can be justified on the similar grounds^9,10^. It was found that the picture based on the KH Hamiltonian can also provide an accurate description of the laser–atom interaction even for low frequencies in the IR range^3,11,12^. Below, we will use the same field parameters and target system as in Ref.^3^. We consider the potassium atom interacting with a strong monochromatic IR field with a frequency $\omega =0.0577$ a.u. corresponding to the wavelength of 800 nm. By its definition (4), the KH Hamiltonian depends only on the parameter $a_0=E_0/\omega ^2$. Therefore, our results will be applicable for other driving laser fields as long as this parameter has the same values.

To describe the field-free potassium atom, we use the model one-electron potential *V*(*r*) given in Ref.^3^. This potential is parametrized to reproduce accurately the bound energies of the 4–6*s*, 4–5*p*, 3–4*d*, 4*f* and 5*g* states. We compute $V_\mathrm{KH}({\varvec{r}})$ by plugging *V*(*r*) into (4) and performing the Gaussian quadrature integration. Because of a singular nature of the integral (4), we use a high order quadrature with 1300 points over the interval $(0,2\pi )$. With $V_\mathrm{KH}({\varvec{r}})$ thus computed, we diagonalize the KH Hamiltonian (3) to find the ground state wave function $\phi _0({\varvec{r}})$ and the corresponding energy $\varepsilon _0$. This diagonalization is performed using the basis set $R_n(r)Y_{l0}(\hat{\varvec{r}})$, where $R_n(r)=Nr^{a_n}e^{-\zeta _n r}$ are the Slater-type orbitals (STO) and $Y_{l0}(\hat{\varvec{r}})$ are the spherical harmonics. We use a set of the so-called split valence STO’s^13^ i.e. we employ the STO’s with different parameters in the exponential functions for each partial wave *l*. Such a split valence basis is known to produce accurate results for various molecular^13^ and atomic^14,15^ Hamiltonians. Use of the different exponents $\zeta _n$ in the STO’s allows us to achieve two important goals. It helps us to cover a large portion of the configuration space, which is necessary if we wish to represent the molecular-like ground state wave-function of the KH atom with its considerable spatial extension. Such a choice also helps us to avoid potential numerical problems due to the near degeneracy of the STO’s, which is often encountered when STO’s with only single $\zeta$-parameter are used for each partial wave. More specifically, for each angular momentum *l* we use a number $N_l$ of the STO’s with $a_n=l+n-1$, $\zeta _n=2/n$ for $n=1,2,\ldots N_l$. The parameter $N_l$ defines the basis size for each partial wave. In the calculations below we used $N_l=12$ for each partial wave.

The calculation is straightforward, and we will not dwell on its numerical details. We choose the quantization direction along the $\hat{x}$ axis. Again, because of a singular nature of the KH potential, the partial wave expansion converges rather slowly so that we take into account the harmonics of the order up to 120. We note that all the necessary convergence checks needed to ensure accuracy of the results that our calculation gives for the ground state eigenvalue of the Hamiltonian (3) have been performed. As an illustration of this statement we mention that if for the field strength of $E_0=0.0534$ a.u. we use harmonics of ranks up to 80 to represent the wave-function and employ the values $N_l=10$ for a number of the STO’s for each *l* we obtain the ground state energy of $-\,0.08214439$ a.u. If, instead, we include in the calculation spherical harmonics of ranks up to 70 with the same $N_l$’s, we obtain the ground state energy of $-\,0.08214438$ a.u. Of a similar magnitude is the norm of the difference of the ground state wave-functions obtained in the two calculations. We have achieved, therefore, a convergence of the order of $10^{-8}$ a.u. with respect to the number of the partial waves included in the calculation. If, on the other hand, we include in the calculation spherical harmonics of ranks up to 70, using $N_l=12$ for each *l*, we obtain the ground state energy of $-\,0.08214596$ a.u., showing that we have achieved a convergence on the level of $10^{-6}$ a.u. with respect to the composition of the radial STO. We can adopt the latter figure as an estimate of the accuracy we may hope to have achieved in representing the ground state of the KH atom.

**Figure 1:**
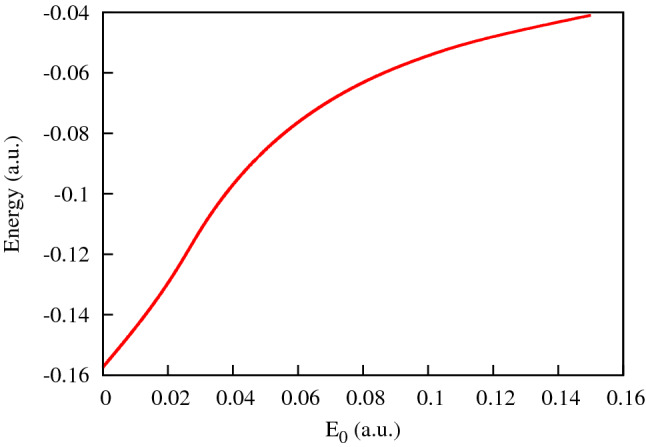
Ground state energy of the KH Hamiltonian (3) for different strengths $E_0$ of the IR driving field.

**Figure 2:**
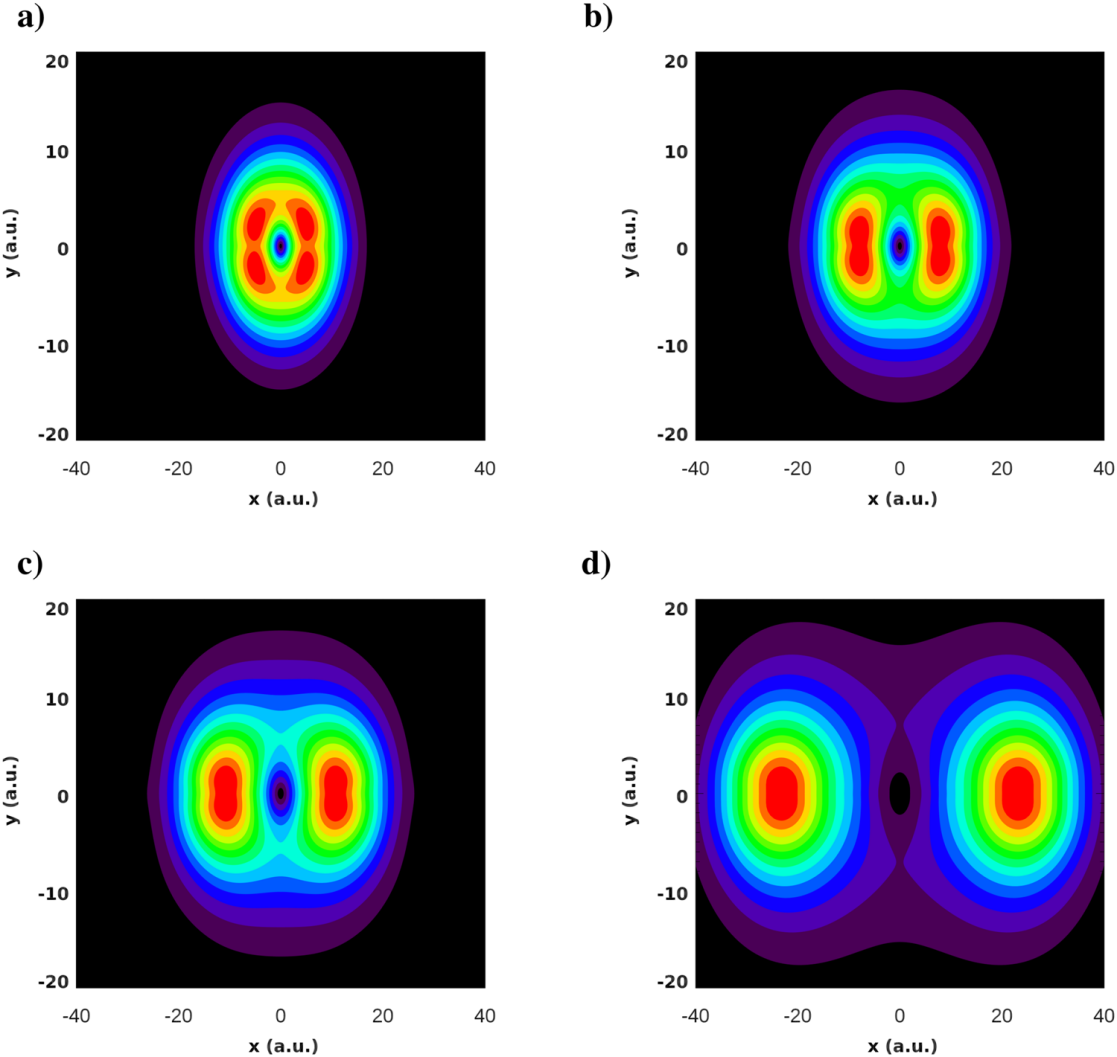
Coordinate density of the ground state wave-function for different values of the IR field strength: $E_0=0.02387$ a.u. (**a**), $E_0=0.04$ a.u. (**b**), $E_0=0.0534$ a.u. (**c**), $E_0=0.1$ a.u. (**d**).

Figures 1 and 2 show, respectively, the ground state energies of the KH Hamiltonian (3) and the coordinate density of the ground state wave function in the (*x*, *y*)-plane for different field strengths of the driving IR field.

As the driving field strength grows, the binding energy $|\varepsilon _0|$ of the KH atom diminishes. At the same time, the coordinate densities in Fig. 2 acquire a distinct character with the two center separation growing approximately as $2a_0$. This should entail certain molecular-like features such as a two-center interference pattern to be present in photoionization from a KH state. We present a study of these effects in the next section.

### Single-photon ionization of KH state

We consider photoionization from the ground state of the KH Hamiltonian (3) by a probe laser pulse with the field *E*(*t*) described by the following expression:

5 $$\begin{aligned} {\varvec{E}}^\mathrm{probe}(t)= {\hat{\varvec{e}}_y}E^\mathrm{probe}_0 \sin ^2{\left( {\pi t\over T_1}\right) }\cos {\Omega t}. \end{aligned}$$

Here $T_1=20T=40\pi /\Omega$ is the total pulse duration, $\Omega$ is the carrier frequency of the probe and $E^\mathrm{probe}_0=0.05$ a.u. is its peak field strength. We consider a weak probe pulse so that the transition amplitude $a_{{\varvec{p}}}$ for ionization into a state with the momentum ${\varvec{p}}$ can be found by using the well-known LOPT expression^16^:

6 $$\begin{aligned} a_{{\varvec{p}}}= -i({\varvec{e}}_y\cdot {\varvec{p}}) \tilde{\phi }_0({\varvec{p}})\tilde{A}^\mathrm{probe}\bigl ({p^2/ 2}- \varepsilon _0\bigr ). \end{aligned}$$

Here $\tilde{\phi }_0({\varvec{p}})$ is the Fourier transform of the ground state wave-function and $\tilde{A}^\mathrm{probe}(x)$ is the Fourier transform of the probe field vector potential. It is assumed in (6) that the final ionized state of the KH Hamiltonian is approximated by a plane wave. Equation (6) also assumes that the velocity gauge is used to describe the interaction of the KH atom and the probe field. We use, therefore, different gauges to describe the atomic interactions with the driving IR and the probe fields, as was done, e.g., in Ref.^5^. We obtain such a description by applying the KH canonical transformation (1) to the Hamiltonian $\hat{H}^\mathrm{V}(t)$ of the system comprising the field-free atom subjected to the driving IR field and the probe field, where both fundamental field $\varvec{A}(t)$ and the probe field $\varvec{A}^\mathrm{probe}(t)$ are initially described using the velocity gauge:

7 $$\begin{aligned} \hat{H}^\mathrm{V}(t)= \hat{H}_\mathrm{atom} + \hat{H}^\mathrm{V}_\mathrm{int}(t), \end{aligned}$$

with $\displaystyle H_\mathrm{atom}= {\hat{\varvec{p}}^2\over 2}+V(r)$ being the field free atomic Hamiltonian and $\hat{H}_\mathrm{int}(t)$ being the velocity gauge interaction Hamiltonian describing atom-field interaction:

8 $$\begin{aligned} \hat{H}^\mathrm{V}_\mathrm{int}({\varvec{r}},t) = \hat{\varvec{p}}\cdot \left( {\varvec{A}}(t)+{\varvec{A}}^\mathrm{probe}(t)\right) . \end{aligned}$$

The transformed Hamiltonian in the KH gauge is then (with operator $\hat{F}(t)$ given by the (1)):

9 $$\begin{aligned} i{\partial F^{\dag }(t)\over \partial t}F(t)+F^{\dag }(t)H^\mathrm{V}F(t)={\hat{\varvec{p}}^2\over 2} + V_\mathrm{atom}\left( {\varvec{r}}- \int \limits _0^t {\varvec{A}}(\tau )\ d\tau \right) + \hat{\varvec{p}}\cdot {\varvec{A}}^\mathrm{probe}(t), \end{aligned}$$

where the first two terms give (for monochromatic field $\varvec{A}(t)$) the Hamiltonian (2), and the part of the Hamiltonian describing atom-probe field interaction remains unchanged since it commutes with the operator $\hat{F}(t)$ in (1) generating the transformation.

## Results and discussion

### Photoelectron momentum distributions

**Figure 3:**
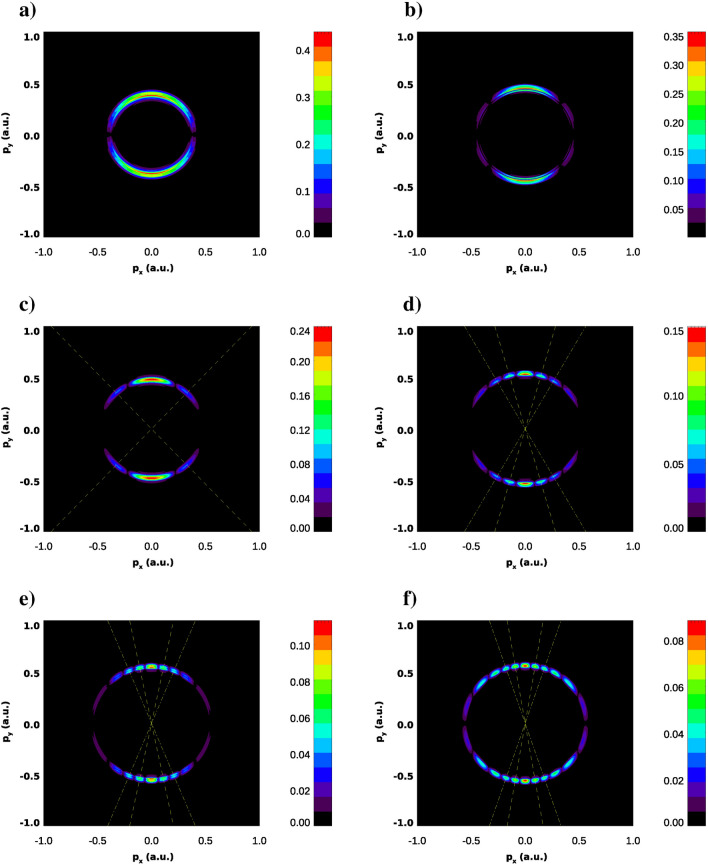
Photoelectron momentum distributions in the (*x*, *y*) plane for various IR field strengths and frequency of the probe field $\Omega =0.2$ a.u. PMD is exponentiated ($P(p_x,p_y)^{1/3}$ is shown) for improving visibility of the patterns. IR field strengths are: $E_0=0.02387$ a.u. (**a**), $E_0=0.04$ a.u. (**b**), $E_0=0.0534$ a.u. (**c**), $E_0=0.1$ a.u. (**d**), $E_0=0.125$ a.u. (**e**), $E_0=0.15$ a.u. (**f**). Lines show directions to low order maxima of the interference pattern.

**Figure 4:**
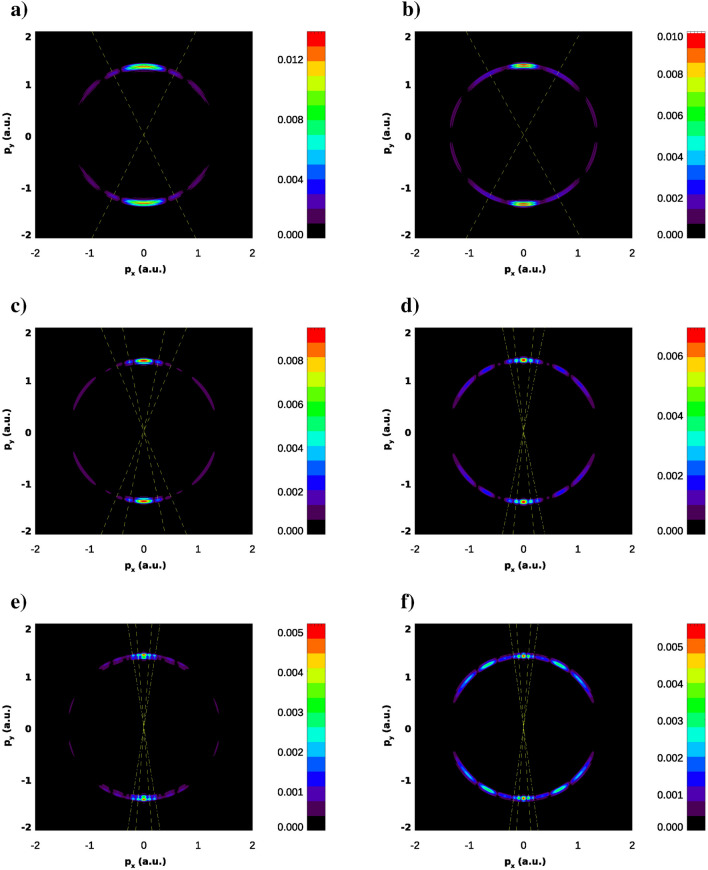
Photoelectron momentum distributions in the (*x*, *y*) plane for various IR field strengths and frequency of the probe field $\Omega =1$ a.u. PMD is exponentiated ($P(p_x,p_y)^{1/3}$ is shown) for improving visibility of the patterns. IR field strengths are: $E_0=0.02387$ a.u. (**a**), $E_0=0.04$ a.u. (**b**), $E_0=0.0534$ a.u. (**c**), $E_0=0.1$ a.u. (**d**), $E_0=0.125$ a.u. (**e**), $E_0=0.15$ a.u. (**f**). Lines show directions to low order maxima of the interference pattern.

In Figs. 3 and 4 we show the photoelectron momenta distributions (PMD) projected on the (*x*, *y*) plane. These results are obtained using the LOPT expression (6) with the KH ground states wave functions prepared as described above. We present results for the two values of the probe field frequency $\Omega =0.2$ a.u. (Fig. 3) and 1 a.u. (Fig. 4) while varying the strengths of the IR field creating the KH state. As expected from the coordinate density plots of Fig. 2, both sets of PMD exhibit interference patterns, albeit not very pronounced. Thus we can attempt to extract some structural information about the KH state from these interference structures.

### Tight binding model

The question that we would like to address first is why the interference patterns are pronounced so poorly even for the large field strengths $E_0$ which correspond to large ‘internuclear’ distances. Indeed, at most, we can observe the main and two secondary interference maxima in Figs. 3 and 4. This is not what we would have expected had we dealt with the case of a genuine two-slit interference in a single photon ionization of a two-center molecule, as suggested by the tight-binding (TBM) or Heitler–London model. In this model (see e.g.^17^), the ground molecular state is represented by a Heitler–London wave function:

10 $$\begin{aligned} \phi _0({\varvec{r}})= [\phi ({\varvec{r}}-{\varvec{R}/ 2}) + \phi ({\varvec{r}}+{\varvec{R}/ 2})]/\sqrt{2}. \end{aligned}$$

**Figure 5:**
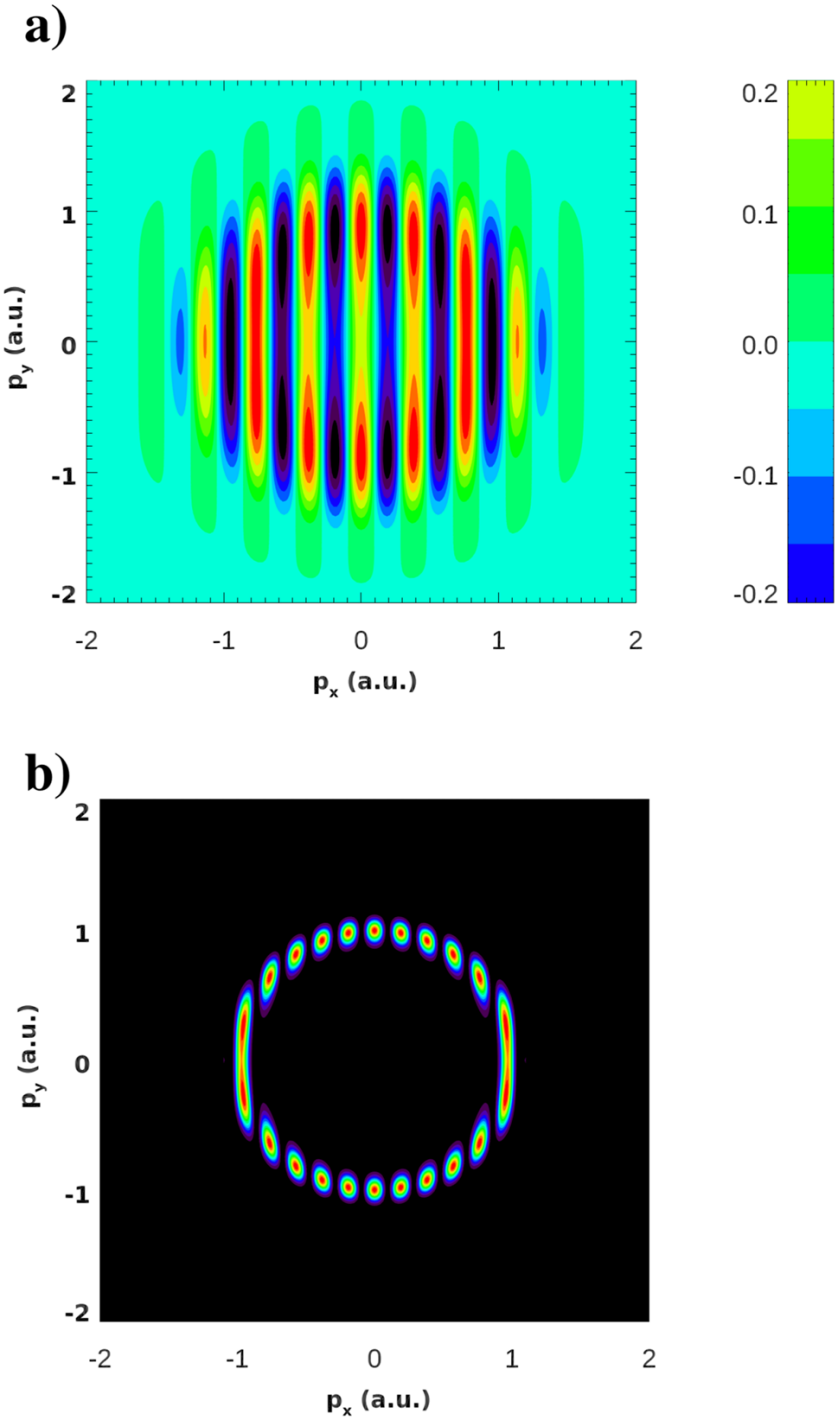
(**a**) The Fourier transform of the wave-function (atomic units are used) and (**b**) the corresponding PMD for the frequency of the probe pulse $\Omega =1$ a.u. for the Heitler-London wave function built form two hydrogen atoms in the ground 1*s* state placed at the distance $R=32.9$ a.u.

In the TBM, the overlap of the two terms is small and $\phi ({\varvec{r}})$ is typically represented by a spherically symmetric atomic-like state. Than, for the Fourier transform of the Heitler–London wave-function (10) we have $\tilde{\phi }_0({\varvec{p}})=\sqrt{2}\tilde{\phi }({\varvec{p}})\cos ({\varvec{p}}\cdot {\varvec{R}}/2)$. This Fourier transform and the corresponding PMD are shown in Fig. 5 for the case where we place two hydrogen atoms in the ground 1*s* state at the distance $R=32.9$ a.u. and the frequency of the probe pulse $\Omega =1$ a.u. The plot in Fig. 5b demonstrates that for a genuine Heitler–London molecule the dependence of the Fourier transform of the wave function on the momenta is dominated for large *R* by a rapidly oscillating cosine factor, leading to the fast oscillations in the PMD in Fig. 5a. The internuclear distance *R* we used in this example is twice the value of the parameter $a_0=E_0/\omega ^2$ for $E_0=0.0534$ a.u. and $\omega =0.057$ a.u. so, basing on the Heitler–London picture, we could expect to see similar PMD for the KH atom in the field range that we consider.

**Figure 6:**
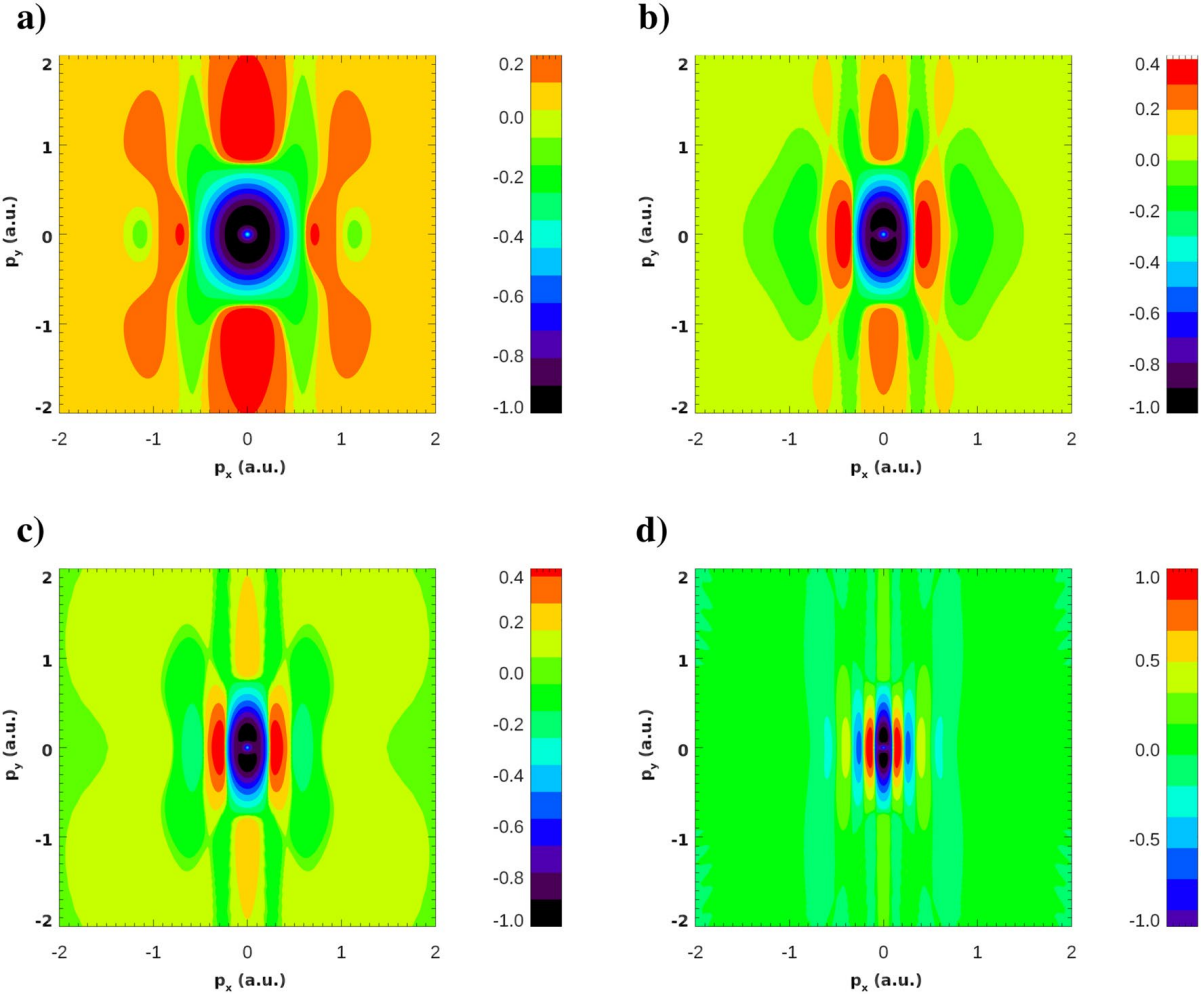
Real part $\Re {(\tilde{\phi }_0({\varvec{p}}))}$ of the Fourier transform of the ground state wave-functions of the KH atom. IR field strengths are: $E_0=0.02387$ a.u. (**a**), $E_0=0.04$ a.u. (**b**), $E_0=0.0534$ a.u. (**c**), $E_0=0.1$ a.u. (**d**). Dimensionless quantity $\left( {\Re {(\tilde{\phi }_0({\varvec{p}}))}\over \mathrm{max}|\Re {(\tilde{\phi }_0({\varvec{p}}))}| }\right) ^{1/3}$ is shown for improving the visibility of the patterns.

The ingredient of (6) which is solely responsible for the numerous interference maxima appearing in the PMD in Fig. 5b) is the Fourier transform $\tilde{\phi }_0({\varvec{p}})$ of the ground state wave function which, in the case of the Heitler–London ground state (10) with a large *R*, is a rapidly oscillating function of the photoelectron momentum. We must conclude, therefore, that for the KH atomic states this is not the case. This can be seen in Fig. 6 where we show the Fourier transforms of the ground state wave functions of the KH atom for different IR field strengths. Figure 6 displays the real part of $\tilde{\phi }_0({\varvec{p}})$). We observe from this figure that the oscillations in the Fourier transforms $\tilde{\phi }_0({\varvec{p}})$ are rapidly decaying in magnitude when we move away from the origin. Thus, in the case of the KH atom, $\tilde{\phi }_0({\varvec{p}})$ is a function which is sharply peaked at the origin. It is this fact that makes only a few interference maxima visible in Figs. 3 and 4. Accordingly, the wave function in the reciprocal coordinate space must be much fuzzier than the Heitler–London wave-function (10) which is sharply peaked at the two centers. A useful measure allowing to characterize this fuzziness of the coordinate wave function and the sharply peaked character of the momentum space wave function is provided by the information theoretic Shannon entropy^18^. The meaning of the entropy that we will use here is quite similar to that in the statistical mechanics. For a given distribution entropy provides a measure of the width of its support. In this respect its meaning is similar to the meaning of the distribution dispersion, but the notion of entropy is more informative since it can be applied for the characterization of distributions with several maxima, such as those displayed in Fig. 2, for which the notion of dispersion is meaningless.

Using the definition of the theoretic Shannon entropy^18^, the respective entropies of the coordinate $S_x(t)$ and momentum $S_v(t)$ distributions can be determined as follows:

11 $$\begin{aligned} S_x(t)= -\int |\Psi (\varvec{r})|^2 \log {|\Psi (\varvec{r})|^2}\ d\varvec{r} \ ,\nonumber \\ S_v(t)= -\int |\tilde{\Psi }({\varvec{p}})|^2 \log {|\tilde{\Psi }({\varvec{p}})|^2}\ d{\varvec{p}}. \end{aligned}$$

Here $\Psi (\varvec{r})$ is the coordinate space wave function describing the system and $\tilde{\Psi }({\varvec{p}})$ is its Fourier transform. Both $\Psi (\varvec{r})$ and $\tilde{\Psi }({\varvec{p}})$ are not dimensionless quantities. So the logarithms of these quantities and consequently the entropies in (11) are defined only up to arbitrary additive constant depending on the units of length we employ. The entropies in (11) share this property with the entropy in classical statistical mechanics^19^), and it is not important as long as we are interested in the entropy change, when this arbitrary additive constant cancels out.

**Figure 7:**
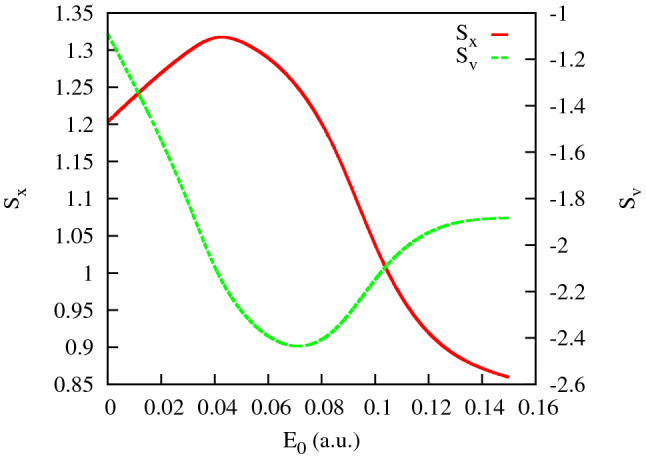
Entropies of the coordinate and momenta distributions for the KH ground state.

The entropies introduced in (11) are shown in Fig. 7. We observe in this figure that, up to the field strengths of $\approx 0.06$ a.u., the coordinate distribution becomes progressively fuzzier, while the momentum distribution is getting sharper. These conditions are quite unfavorable to forming an interference pattern. For higher field strengths, these trends are reversed. The momentum distribution is becoming a little ‘wider’, thus enabling the formation of an interference pattern. We can draw the same conclusions from the figures above showing the PMD’s. For the higher field strengths we do see the interference patterns staring to develop in the PMD’s. First and second order interference maxima are clearly seen, although they are not exactly at the places prescribed by the TBM. The cosine factor in the TBM positions the interference maxima at $p_x R=2\pi n$, with $n=0, \pm 1 \ldots$. Since the electron energy is fixed by the energy conservation relation $p_x^2+p_y^2= 2\varepsilon _0+ 2\Omega$ these two relations determine the position of interference maxima in the $(p_x,p_y)$-plane once *R* is known. In Figs. 3 and 4, we display the rays at the locations of the $n=\pm 1$ and $n=\pm 2$ interference maxima by choosing the value of *R* so that it gives us the exact direction to the first order maximum of the PMD. As one can see, the locations of the maxima with $n=\pm 2$ are not very well reproduced, so we are still not quite in the region of applicability of the TBM even for the two-center distances as large as 40 a.u. (the value we have in Fig. 2 for $E_0=0.1$ a.u.).

**Figure 8:**
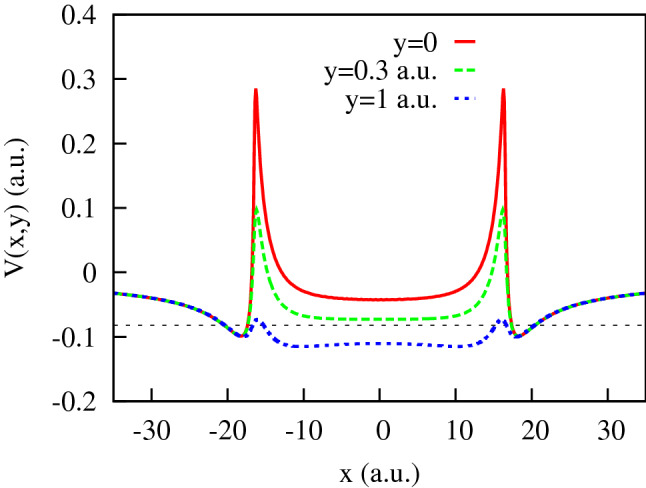
Cuts of the KH potential *U*(*x*, *y*) for different *y* for the IR field strength $E_0=0.0534$ a.u.

### Peculiar features of the KH potential

**Figure 9:**
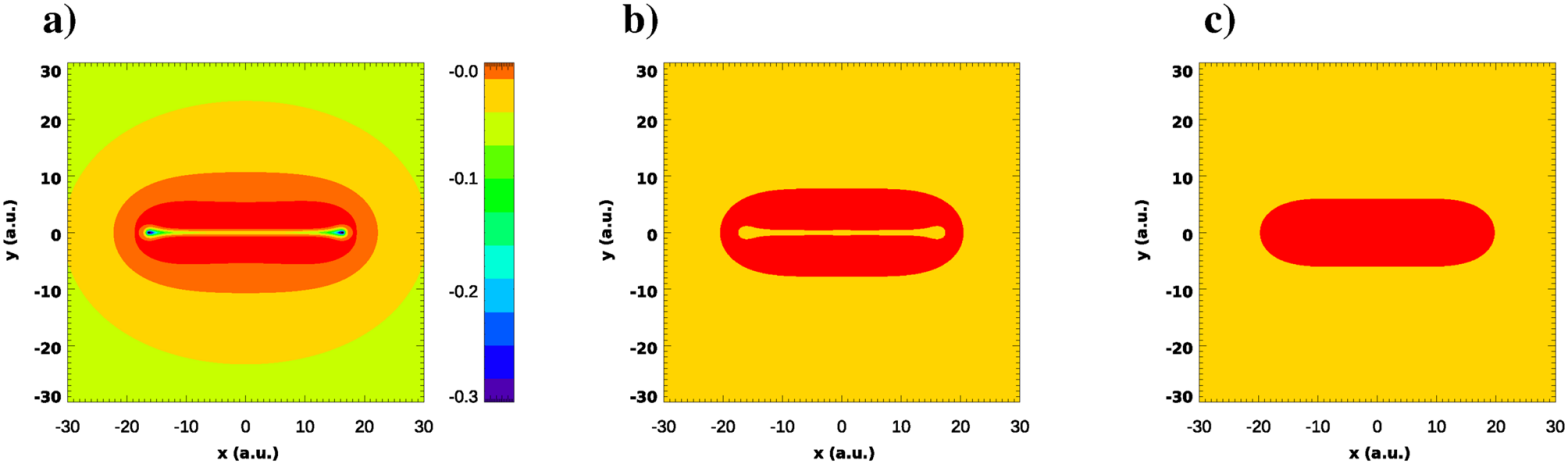
(**a**) The electron kinetic energy $T=E-V(x,y)$ (a.u.) in the KH atom for the IR field strength $E_0=0.0534$ a.u. (**b**) Classically allowed area (in red) in the (*x*, *y*)-plane for $E_0=0.0534$ a.u. (**c**) Classically allowed area (in red) in the (*x*, *y*)-plane for the same field parameters and atomic Coulomb potential.

To understand why even for such large two-center distances the TBM is still a poor approximation, we display in Fig. 8 the cuts of the KH potential *U*(*x*, *y*) for different *y*. One can see from this figure that electron tunneling from one center to another one along the two-center axis has a vanishingly small probability, the barrier being forbiddingly high. If, however, we move only very slightly away from the two-center axis, the barrier lowers dramatically or disappears altogether. This feature of the KH potential can be illustrated better yet if we plot the electron kinetic energy, i.e, the quantity $T(x,y) = E-V(x,y)$ in the (*x*, *y*)-plane. This is done in Fig. 9a. The regions in the (*x*, *y*)-plane with $T>0$ are classically allowed. This classically allowed area (CAA) in the (*x*, *y*)-plane is shown (in red) in Fig. 9b. One can observe that there is a broad CAA in the *x*, *y*-plane which connects both potential wells of the KH potential barrier situated at $x= \pm \, a_0$. This connected character of the CAA makes it easy for the electron to travel out from the KH potential well, thus smearing the coordinate space wave function between the two wells. The notable exception is the high ridge along the two-center axis where the tunneling barrier is still very high. It is this feature of the KH potential, which is absent in real diatomic molecules, that ultimately makes the electron wave function so fuzzy in the coordinate space. That, in turn, makes the TBM a poor approximation even for very large two-center distances. The wave function in the reciprocal momentum space is sharply peaked near the origin exhibiting not too many oscillations leading, therefore, to the PMD’s with only low order interference peaks present. We shall see, nevertheless, that even these low-order interference patterns can provide reliable information about the spatial structure of the KH state.

This discussion of the properties of the CAA of electron’s motion was based on the connected character of the CAA for a particular KH potential resulting from the potential function *V*(*r*) we employ in the present work. The general pattern of the CAA and hence our conclusions would not change had we used another potential function. As an illustration of this statement, we show in Fig. 9c the CAA for the KH potential arising from the Coulomb $V(r)=-1/r$ potential for the same field parameters. As one can see from the figure, the CAA in the case of the Coulomb atomic potential is a singly connected region in the (*x*, *y*)-plane, while for the potential function *V*(*r*) we considered above, this is not the case, a ridge in the KH potential appears due to the strongly-repulsive feature present in the potential function *V*(*r*) introduced in^3^ that we used above. This difference is not very important, however, essential point is that in the Coulomb case the CAA is also a connected region in the (*x*, *y*)-plane.

### Analysis of the spatial structure of the KH states

**Figure 10:**
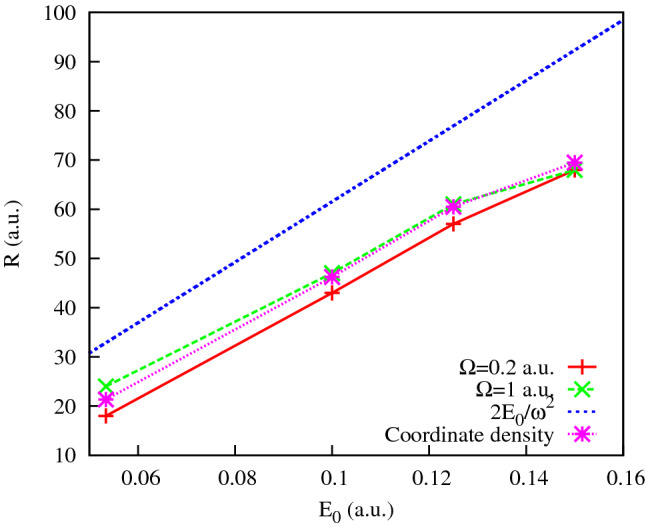
‘Internuclear’ distances obtained from the interference patterns in Figs. 3 and 4.

In Fig. 10 we show estimates for the ’internuclear’ distance *R* we obtain from the interference structures in the PMD’s in Figs. 3 and 4. These structures are visible in Figs. 3 and 4 distinctly for the IR field strength of 0.0534 a.u. and higher. The estimates were obtained as described above by adjusting the ‘internuclear’ distance *R* so that the two-slit interference relation of the TBM and the energy conservation equation give us the correct locations of the first order maxima in the PMD. We compare results for *R* thus obtained with the naive estimate $R=2a_0$ and the estimate we obtain from the coordinate density plots like the ones shown in Fig. 2. These “coordinate density” estimates for *R* are obtained simply as locations of the maxima of the coordinate density in the (*x*, *y*)-plane. As one can see from Fig. 10, we obtain pretty good (better than 10%) agreement between the two values of *R* if we use the probe pulse with $\Omega$=1 a.u. for all the field strengths that we consider. For the probe frequency $\Omega =0.2$ a.u. the agreement is worse but still quite acceptable. More importantly, agreement between the values of *R* inferred from the interference pattern in the PMD’s and the value obtained by the analysis of the coordinate density of KH states improves with increasing IR field strength. One may hope, therefore, that such estimates of the ‘internuclear’ distance *R* can be used for the purposes of accurate calibration of strong laser fields. This approach relies only on the atomic property and the laser intensity at the position of the atom. Thus, it would provide a more accurate way to calibrate the absolute intensity of the laser beam than the methods relying on the macroscopic property of the laser beam and ionization models^20,21^.

### Preparation and observation of KH states

We have considered so far the idealized case of a KH state interacting with an XUV pulse, not touching upon the question of how such a state can be created and how (and if) the interference patterns we described above can be observed in the real experiment. This issue is somewhat tangential to the main topic of the manuscript, which was to illustrate the not quite molecular character of KH states. Nevertheless, this issue is, of course, of great importance, since without a possibility of an experimental observation the findings we reported above can have only a purely academic interest.

To elucidate this issue we performed a series of time-dependent Schrödiger equation (TDSE) calculations, solving the TDSE:

12 $$\begin{aligned} i {\partial \Psi ({\varvec{r}},t) \over \partial t}= \hat{H}^\mathrm{V}(t) \Psi ({\varvec{r}},t), \end{aligned}$$

using the velocity gauge Hamiltonian (7) with interaction Hamiltonian given in (8). The form of the functions describing the vector potentials in (8) was chosen as follows:

13 $$\begin{aligned} {\varvec{A}}(t)= & {} -{\hat{\varvec{e}}_x}{E_0\over \omega } \sin ^2{\left( {\pi t\over T_1}\right) }\sin {\omega t}, \nonumber \\ {\varvec{A}}^\mathrm{probe}(t)= & {} -f(t){\hat{\varvec{e}}_y}{E_0^\mathrm{probe}\over \Omega } \sin {\Omega (t-t_m)}. \end{aligned}$$

In (13) the total duration of the fundamental pulse was $T_1= 25 T$ where $T=2\pi /\omega$ is an optical cycle corresponding to the fundamental pulse base frequency $\omega$, $t_m=T_1/2$ is the midpoint of the fundamental pulse. The total duration of the probe pulse was twenty optical cycles: $T_2=40\pi /\Omega$, and for the envelope *f*(*t*) of the probe pulse we used an expression: $\displaystyle f(t)= \cos ^2{\left( {\pi (t-t_m)\over T_2}\right) }$ if $|t-t_m|\le T_2/2$ and $\displaystyle f(t)=0$ otherwise.

#### IR fundamental field

**Figure 11:**
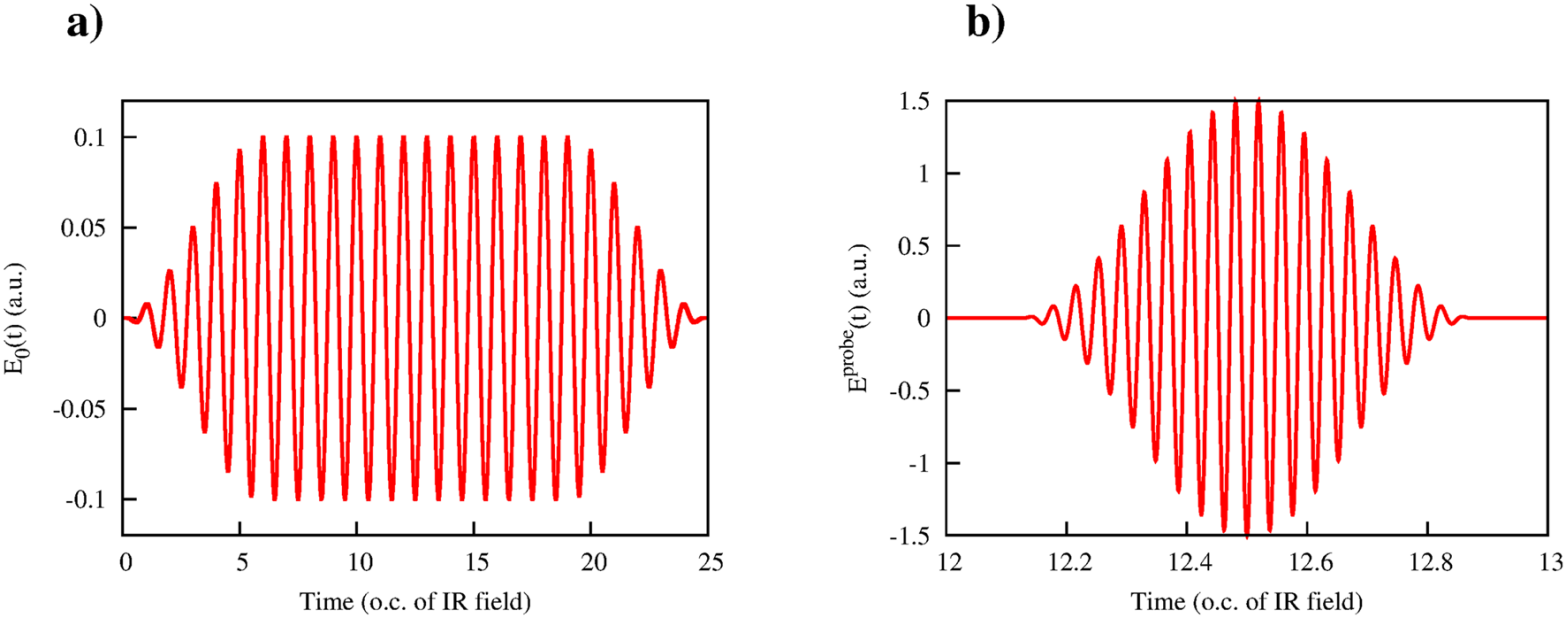
$x-$ component of the fundamental IR field (**a**) and $y-$component of the XUV field (**b**) used in the TDSE calculation. IR field parameters: $E_0=0.1$ a.u., $\omega =0.0577$ a.u. XUV field parameters: $E_0^\mathrm{probe}= 1.5$ a.u., $\Omega =1.5$ a.u.

We consider first the case of the IR fundamental field, with the fields parameters chosen as follows: $E_0=0.1$ a.u., $\omega =0.0577$ a.u. for the fundamental field, and $E_0^\mathrm{probe}= 1.5$ a.u., $\Omega =1.5$ a.u for the probe field. Electric fields of the pulses are shown in Fig. 11. The TDSE (12) was solved numerically using the procedure we described in detail elsewhere^22,23^. We will give, therefore, only its brief description. The solution to (12) is represented as:

14 $$\begin{aligned} \Psi ({\varvec{r}},t)= \sum \limits _{l,m}^{l_\mathrm{max}} f_{lm}(r,t) Y_{lm}(\theta ,\phi ), \end{aligned}$$

where $Y_{lm}(\theta ,\phi )$ are spherical harmonics, radial functions $f_{lm}(r,t)$ are defined on the points of a grid with the step-size $\delta r=0.1$ a.u. in a box of the size $R_\mathrm{max}$. A system of the coupled equations for the radial functions $f_{lm}(r,t)$ resulting from the substitution of the expansion (14) into the TDSE was propagated in time on the interval (0, 25*T*) (here *T* is the optical cycle of the fundamental field) using the matrix iteration method^24^. The initial state for the time-propagation was the ground state of the potential *V*(*r*) we used above. As previously, we use the coordinate system with the quantization axis in the *x*-direction. Transitions caused by the fundamental field $\varvec{A}(t)$ conserve then the magnetic quantum number *m* in the expansion (14). On the other hand, because of the well-known dipole selection rules, absorption or emission of a probe photon leads to a change of *m* by one unit. If we start with the $m=0$ initial state, and we are interested in a single probe photon absorption or emission processes we can, therefore, restrict the sum in *m* in (14) to $m=0,\pm \, 1$. As for the parameter *l* in this expansion it was restricted to the range $l\le l_\mathrm{max}$. The values of the parameters $l_\mathrm{max}$ and the parameter $R_\mathrm{max}$ defining the radial box-size were chosen after the convergence checks as: $l_\mathrm{max}=60$, $R_\mathrm{max}=4000$ a.u. As an illustration of the level of convergence we achieve, we mention that choosing the parameters $l_\mathrm{max}=60$ and $R_\mathrm{max}=4000$ we obtain total ionization probability of 0.867092 after the end of the fundamental pulse. Repeating the calculation with $l_\mathrm{max}=50$ changes this value to 0.867096.

**Figure 12:**
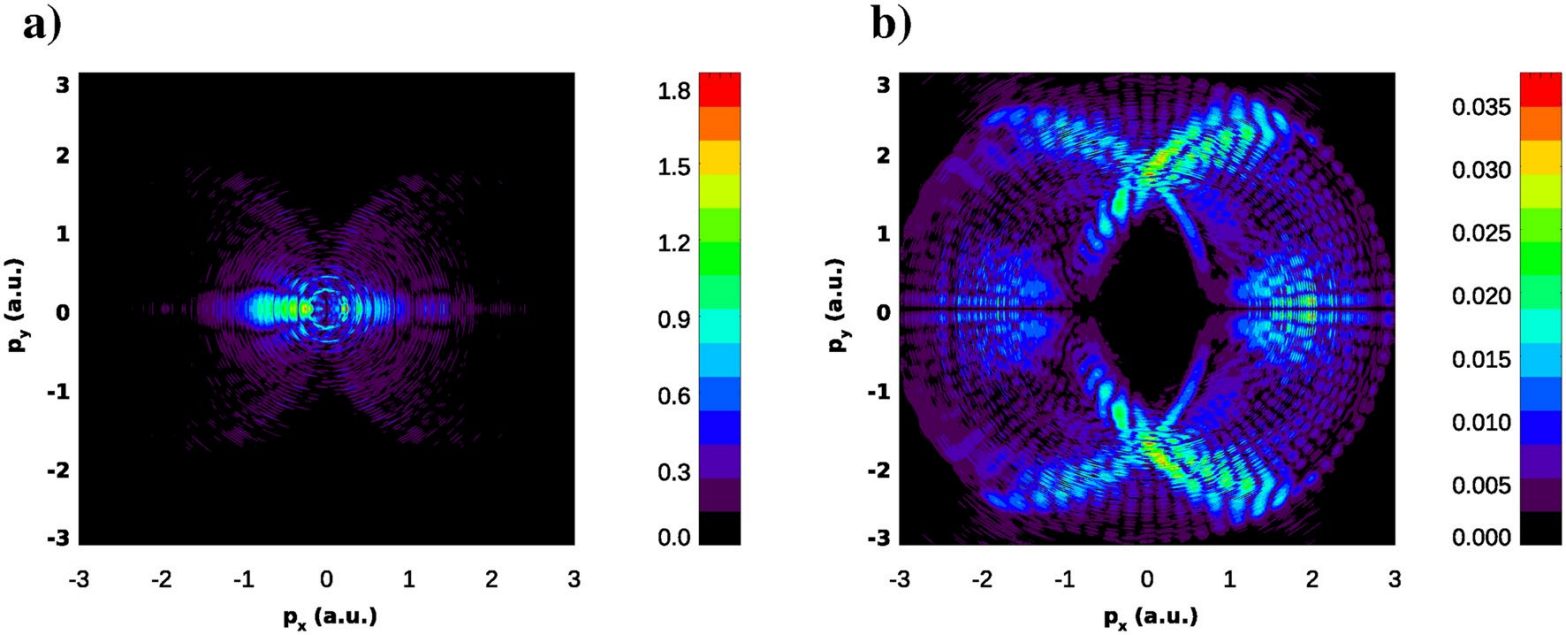
Unfiltered (**a**) and filtered (**b**) PMD in the (*x*, *y*) plane. PMD is exponentiated ($P(p_x,p_y)^{1/3}$ is shown) for improving visibility of the patterns. IR field parameters: $E_0=0.1$ a.u., $\omega =0.0577$ a.u. XUV field parameters: $E_0^\mathrm{probe}= 1.5$ a.u., $\Omega =1.5$ a.u.

We compute ionization amplitude by projecting the wave-function at the end of the fundamental pulse on the set of the (ingoing) scattering states of the potential *V*(*r*): $\displaystyle a({\varvec{p}})= \langle \phi _{{\varvec{p}}}^{-}|\Psi (T_1)\rangle$. The PMD thus obtained is shown in Fig. 12a. It presents a very complex picture. IR field plays the dominant role in the ionization process which makes it difficult to single out the effects of the XUV field ionization which we are looking for. A ‘filtering’ procedure allowing to separate combined IR and XUV field effects is needed. The procedure employed in Ref.^5^, based on the energy separation of the two contributions to the PMD does not work in the present case. We can apply, instead, the following two-step filtering procedure. As we mentioned above, for the geometry we employ, with perpendicular polarization directions of the two fields, absorption of an IR photon leaves the angular momentum projection on the *x*- axis unchanged, while absorption of an XUV photon changes this projection by one unit. If, therefore, in the expression for the wave-function (14) at the end of the pulse, we leave only terms with $m=\pm \, 1$, we effectively single out from the wave-function the component describing processes in which one XUV photon and any number of IR photons have been absorbed or emitted. This procedure can be alternatively regarded as a PMD measurement accompanied by an additional requirement that in addition to the electron’s momentum, we measure at the detector *x*-projection of the electron’s angular momentum in the final state and require it to be $m=\pm \, 1$. Note, that, though operators $\hat{\varvec{p}}$ and $\hat{l}_x$ do not commute, such a measurement is possible in principle. Indeed, the well-known quantum-mechanical relation for the uncertainties which non-commuting variables $l_x$ and $p_i$ (where *i* may stand for *x*, *y*, or *z*) represented by operators $\hat{l}_x$ and $\hat{p}_i$ have in a state $|\Psi (T_1)\rangle$ reads^25^: $\displaystyle \Delta l_x \Delta p_i \ge {1\over 2} \bigg |\bigg \langle \Psi (T_1)|[\hat{l}_x,\hat{p}_i]|\Psi (T_1)\bigg \rangle \bigg |$, where $\Delta l_x$ and $\Delta p_i$ are the uncertainties (dispersions) of the variables $l_x$ and $p_i$ in the state $|\Psi (T_1)\rangle$. If, as we do, we measure electron’s momentum in the (*x*, *y*)-plane, then $[\hat{l}_x,\hat{p}_x]=0$ and $\langle \Psi (T_1)|[\hat{l}_x,\hat{p}_y]|\Psi (T_1)\rangle =i\langle \Psi (T_1)|\hat{p}_z|\Psi (T_1)\rangle =0$ (because of the symmetry of the fields configuration).

**Figure 13:**
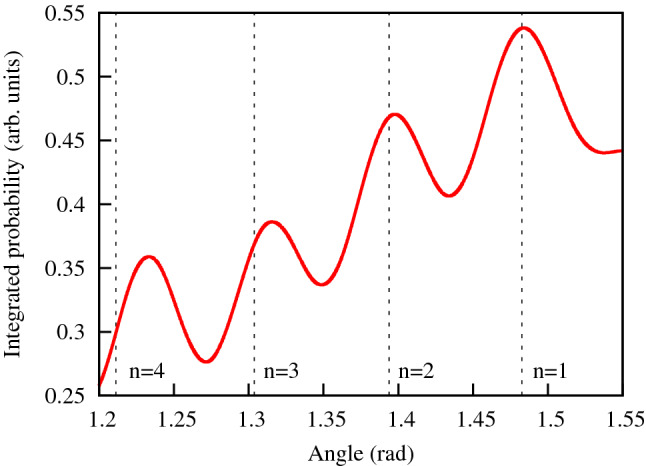
Radially integrated filtered PMD. IR field parameters: $E_0=0.1$ a.u., $\omega =0.0577$ a.u. XUV field parameters: $E_0^\mathrm{probe}= 1.5$ a.u., $\Omega =1.5$ a.u.

The PMD $P^\mathrm{filter}(p_x,p_y)$ thus filtered is shown in Fig. 12b. It presents still a rather complex picture. The second stage of the filtering procedure we use consists in computing radially integrated distribution which, after introducing polar coordinates $p,\phi$ in the $(p_x,p_y)$-plane can be computed as an integral $\displaystyle Q(\phi )=\int P^\mathrm{filter}(p_x,p_y) dp$. This radially integrated distribution is shown in Fig. 13. Dotted lines in the figure are the rays pointing at the interference maxima in the $p_x,p_y$-plane. As above, we define these rays using the interference formula $p_x R=2\pi n$ and the energy conservation relation $p_x^2+p_y^2= 2\varepsilon _0+ 2\Omega$, where we use the same value $\varepsilon _0= -\,0.05434$ a.u. that we obtained above for the ground state energy of the KH-state at $E_0=0.1$ a.u. The dotted lines in the Fig. 12 are obtained by using the value $R=42$ a.u. in the interference relation. We see that for this value of the ‘internuclear’ distance *R* the maxima of the radially integrated probability in Fig. 12 reproduce satisfactorily the low order interference maxima. This value of *R* is quite close to the values of *R* for $E_0=0.1$ a.u. shown in Fig. 10 we obtained above by analyzing perturbatively photo-ionization of the KH states. We see, thus, that the result we obtain from the TDSE calculation describing realistic experimental setup, where atom is driven by the fundamental IR field and the XUV field, and the result we obtain by studying ‘ideal’ situation of a one-photon ionization of the ground state of the KH potential, give similar results.

#### XUV fundamental field

The PMD we obtained above from the TDSE solution are rather complex, so we had to rely on a rather elaborate procedure to extract information from the spectra. A much simpler picture is obtained if the fundamental field is also in the XUV domain. As we mentioned above, the energies and the wave-functions of the KH states depend only on the combination $a_0=E_0/\omega ^2$ of the fundamental field. If, therefore, we use larger $\omega$ for the fundamental field and scale up the field amplitude $E_0$ so that the parameter $a_0$ has the same value as for the KH LOPT calculations presented in Fig. 4, we could directly compare the results of the TDSE and the KH LOPT calculations.

For the TDSE calculations we use the following fields parameters: $\omega =0.5$ a.u. for the fundamental field, $E_0^\mathrm{probe}= 0.05$ a.u., $\Omega =1$ a.u for the probe field. Pulse shapes and durations (expressed in units of an optical cycle corresponding to the base frequency) are the same as in the TDSE calculation for the IR fundamental field we described above and are given by the (13). TDSE was solved using the same numerical procedure we described in the previous section. We report below results for three values of the fundamental field peak field strength: $E_0=1.84$ a.u., $E_0=3.07$ a.u., and $E_0=4.11$ a.u. Parameter $a_0$ for these peak field strengths has the same values as for the field strengths of 0.02387 a.u., 0.04 a.u., 0.0534 a.u., and base frequency $\omega =0.057$ a.u., thus enabling comparing of the results with the results shown in Fig. 4.

**Figure 14:**
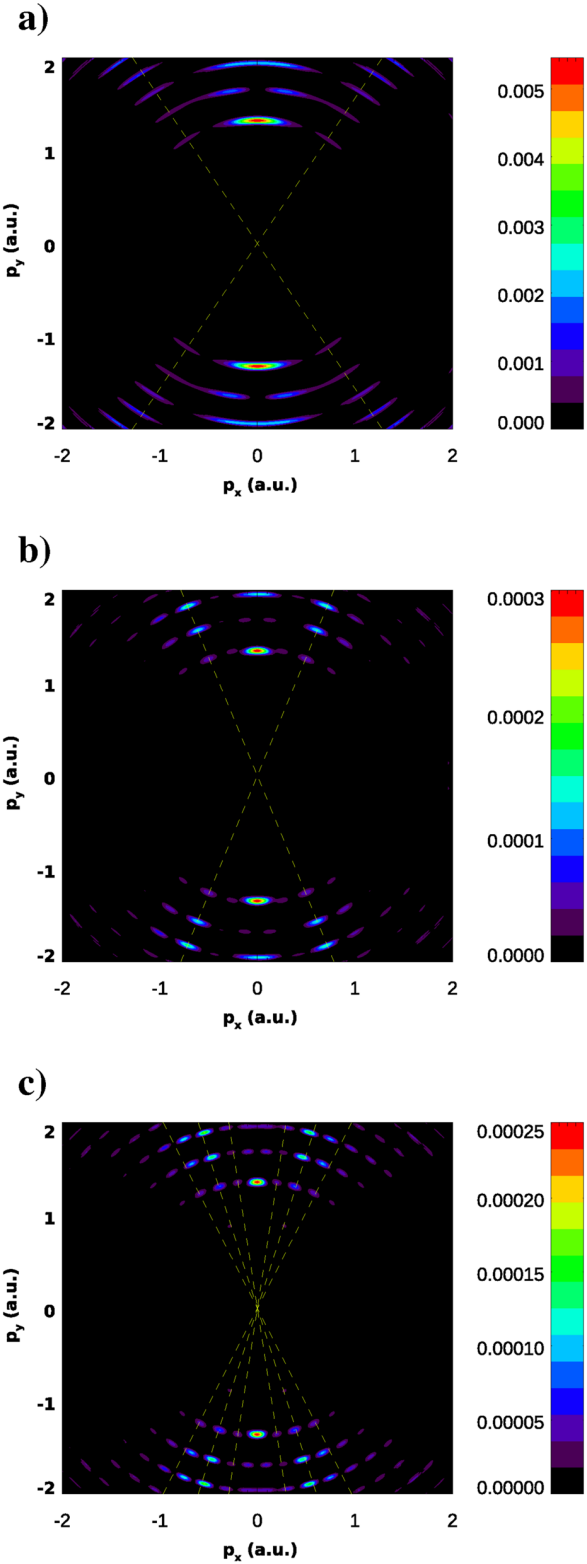
Filtered photoelectron momentum distributions in the (*x*, *y*) plane for various field strengths $E_0$ and frequency $\omega =0.5$ a.u. of the fundamental field. Fundamental field strengths are: $E_0=1.84$ a.u. (**a**), $E_0=3.07$ a.u. (**b**), $E_0=4.11$ a.u. (**c**). Probe field parameters: $E^\mathrm{probe}=0.05$ a.u., $\Omega =1$ a.u. Lines show directions to low order maxima of the interference pattern.

The filtered PMD’s for these field parameters are shown in Fig. 14. We used the same filtering procedure we described above, i.e. we projected out of the wave-function the component which describes processes with participation of only one probe photon. The filtered spectra look much neater that in the case of the IR fundamental field, and we can extract some information from them without the necessity of using supplementary procedures, such as the radial integration we used above in the case of the IR fundamental field. We see a distinct set of rings, which correspond to absorption of additional photons form the fundamental field. The rings with smallest radii, as one can see by comparing PMD in Figs. 4 and 14, are the ones which are of interest to us. Just as we did in Fig. 4, we draw lines through the interference maxima and determine the ‘internuclear’ distances from the two relations we used above: the interference criteria, and the energy conservation formula, where we use $\Omega =1$ a.u., and values for the ground state energies of the KH states for the corresponding value of the parameter $a_0$. Results for the ‘internuclear’ distances we obtain in this way are shown in the Table 1 below.

**Table 1:** ‘Internuclear distance’ R (a.u.) from KH LOPT and TDSE with XUV fundamental field calculations.

$a_0$ (a.u.)	KH LOPT	TDSE with XUV fundamental
7.35	9.5	8.8
12.31	11	12.8
16.44	24	28.2

One can see that the numbers in the second column of the Table 1, representing the KH LOPT calculations (Fig. 4), and the third column of the Table, showing the results of the TDSE with XUV fundamental field calculations (Fig. 14) agree reasonably well for the same values of the parameter $a_0$.

## Conclusion

We conduct a systematic investigation of a Kramers–Henneberger state of the potassium atom subjected to an IR dressing field and probed by an XUV pulse. The KH atom thus formed displays a characteristic two-center charge density pattern in the coordinate state which resembles that of a diatomic homo-nuclear molecule. Single-photon ionization of such a state with an XUV probe produces the photoelectron momentum distribution with characteristic two-center interference maxima. Surprisingly, only lowest order interference maxima can be seen in the PMD of the KH atom that differentiates it strongly from a diatomic molecule of a comparable spatial extent. We explain this phenomenon by analyzing the electron localization in the KH atom in the coordinate and reciprocal momentum spaces. Except for the two-center axis where the KH potential well is rather sharp, the electrons can travel easily from their localization centers thus making the coordinate space wave function of the KH atom rather fuzzy. The respective momentum space wave function peaks very sharply near the origin which hardens formation of interference maxima in the PMD. These findings place certain restrictions on the direct analogy between the KH atomic state and a homo-nuclear diatomic molecule that was suggested earlier in Ref.^5^. We show, that the low order interference pattern which can be observed in the PMD’s can be used to retrieve accurate information about spatial dimension of the KH state. This information can be employed, in principle, for an accurate calibration of the high intensity electromagnetic fields. As the results from Fig. 7 show, the momentum entropy slowly increases with the IR electric field strength. Accordingly, the support of the momentum space wave function grows, and it becomes less sharply concentrated near the origin, making conditions for the observation of the interference maxima progressively more favorable for higher field strengths. We see, of course, the same tendency in the PMD’s shown in Figs. 3 and 4. The field calibration procedure based on the determination of *R* as a function of the field strength may prove, therefore, progressively more accurate with increasing electric field.

For this procedure to become a realistic tool of measuring field intensities, a number of obstacles are to be overcome. KH states have been observed in the experiment. Perhaps the most direct and striking manifestation of the role of the KH states is the process of the laser driven stabilization^2^. Experimental evidence for this process was presented decades ago in Ref.^26^. Another experimental confirmation of the role played by KH states in laser-matter interactions came from the experiments on acceleration of neutral atoms in strong fields^27^. A thorough analysis^9^ of the experimental data shows that much better fit to the experimental data is obtained if accelerated atoms are assumed to be excited into the KH and not into the Rydberg atomic states. On the other hand, finding in the experiment a signature of the KH states in the PMD’s may not be an easy task. We saw an illustration of the difficulty above. In the case of the IR fundamental field, that is important in practice, the PMD’s are dominated by the ionization induced by the IR field, which makes it difficult to separate effects of the IR and XUV fields. We devised a rather elaborate theoretical procedure based on the simultaneous detection of electron’s momentum and projection of the angular momentum. As we noted, for the fields geometry we employ such a measurement is possible in principle, though it not yet clear how to realize it experimentally. It could, perhaps, be implemented by using magnetic field to separate different angular momentum projections. Another obstacle, which is, however, easier to overcome is the symmetry breaking of Kramers-Henneberger atoms by the ponderomotive force. As we noted above, KH atoms in a linearly polarized homogeneous electric field exhibit the structure of “dichotomy”. In an experimental situation, where a focused laser beam is used, electrons experience the ponderomotive force which breaks the symmetry of the KH states^28^. For the intensity range we consider in the manuscript this effect can be controlled in practice by using not too small beam size. For example, for the intensity of $3.5 \times 10^{14}$ W/cm$^2$, pulse FWHM of 10 fs and beam size of 20 $\upmu$m, an electron’s coordinate shift due to the ponderomotive force is of the order of 0.1 a.u. of length, which is much smaller than the ‘internuclear’ distance at this intensity, which is of the order of 50 a.u.

## Data Availability

The datasets used and/or analysed during the current study available from the corresponding author on reasonable request.
